# Antimicrobial and Biocide Resistance among Feline and Canine *Staphylococcus aureus* and *Staphylococcus pseudintermedius* Isolates from Diagnostic Submissions

**DOI:** 10.3390/antibiotics11020127

**Published:** 2022-01-19

**Authors:** Andrea T. Feßler, Anissa D. Scholtzek, Angela R. Schug, Barbara Kohn, Christiane Weingart, Anne-Kathrin Schink, Astrid Bethe, Antina Lübke-Becker, Stefan Schwarz

**Affiliations:** 1 Institute of Microbiology and Epizootics, Centre for Infection Medicine, Department of Veterinary Medicine, Freie Universität Berlin, 14163 Berlin, Germany; anissa.scholtzek@bfr.bund.berlin (A.D.S.); a.schug@posteo.de (A.R.S.); anne-kathrin.schink@fu-berlin.de (A.-K.S.); astrid.bethe@fu-berlin.de (A.B.); antina.luebke-becker@fu-berlin.de (A.L.-B.); 2 Veterinary Centre for Resistance Research (TZR), Freie Universität Berlin, 14163 Berlin, Germany; barbara.kohn@fu-berlin.de (B.K.); christiane.weingart@fu-berlin.de (C.W.); 3 Unit Bacterial Toxins, Food Service, Department Biological Safety, German Federal Institute for Risk Assessment, 10589 Berlin, Germany; 4 Small Animal Clinic, Department of Veterinary Medicine, Freie Universität Berlin, 14163 Berlin, Germany

**Keywords:** dog, cat, infections, *S. aureus*, MRSA, *S. pseudintermedius*, MRSP, antimicrobial resistance, biocide susceptibility

## Abstract

A total of 114 *Staphylococcus* isolates from various infections of companion animals, including 43 feline *Staphylococcus aureus*, 19 canine *S. aureus*, 11 feline *Staphylococcus pseudintermedius* and 41 canine *S. pseudintermedius* were investigated for (i) their susceptibility to 24 antimicrobial agents and three combinations of antimicrobial agents by broth microdilution following CLSI recommendations and (ii) the corresponding resistance genes. In addition, the isolates were tested for their susceptibility to the four biocides benzalkonium chloride, chlorhexidine, polyhexanide and octenidine by a recently developed biocide susceptibility testing protocol. Penicillin resistance via *blaZ* was the dominant resistance property in all four groups of isolates ranging between 76.7 and 90.9%. About one quarter of the isolates (25.4%) proved to be methicillin-resistant and carried the genes *mecA* or *mecC*. Macrolide resistance was the second most prevalent resistance property (27.2%) and all isolates harbored the resistance genes *erm*(A), *erm*(B), *erm*(C), *erm*(T) or *msr*(A), alone or in combinations. Fluoroquinolone resistance was detected in 21.1% of all isolates tested, whereas tetracycline resistance via *tet*(K) and/or *tet*(M) occurred in 19.3% of the isolates. Resistance to last resort antimicrobial agents in human medicine was seen only in single isolates, if at all. The minimal inhibitory concentrations (MICs) of the four biocides showed unimodal distributions and were very similar for the four groups of staphylococci. Because of the large number of (multi)resistant isolates, antimicrobial susceptibility testing of feline and canine *S. aureus* and *S. pseudintermedius* isolates is highly recommended before the start of an antimicrobial chemotherapy. Moreover, no hints towards the development of biocide resistance were detected.

## 1. Introduction

Staphylococci, especially *Staphylococcus* (*S*.) *aureus* and *S. pseudintermedius*, are often involved in a wide range of infections among dogs and cats [1,2]. Multiresistant canine and feline *S. aureus* and *S. pseudintermedius* isolates may also pose a risk to human health [3,4,5]. Companion animals, such as dogs and cats, which harbor these multiresistant staphylococci, often live in close contact to their owners and can transfer these pathogens via direct contact or contamination of the household environment to humans [5,6,7]. A recent systematic meta-analysis suggested that contact with pet animals is a risk factor for the acquisition of methicillin-resistant *S. aureus* (MRSA) [8].

Efficient treatment of staphylococcal infections commonly requires the use of antimicrobial agents. As a consequence of the selection pressure imposed by the use of antimicrobial agents, staphylococci have developed and/or acquired resistances to virtually all classes of antimicrobial agents [9,10,11]. This is also true for the two staphylococcal species, *S. aureus* and *S. pseudintermedius* [9,12]. In particular, methicillin resistance has emerged as a major resistance property in both pathogens during the last decade worldwide [9,12,13,14,15,16,17,18,19,20,21,22]. By the definition of the Clinical and Laboratory Standards Institute (CLSI), methicillin-resistant staphylococci are considered resistant to all β-lactams approved for veterinary use [23]. To worsen the situation, both MRSA and methicillin-resistant *S. pseudintermedius* (MRSP) are usually also resistant to a number of non-β-lactam antimicrobials, thereby further reducing the therapeutic options. Two multicenter studies have confirmed this observation for MRSP isolates from dogs [17] and cats [18] obtained from clinical samples in European countries and North America. In these studies, a wide range of antimicrobial resistance genes have been detected. Besides resistance genes commonly found in staphylococci, rarely detected antimicrobial resistance genes, such as the gene *ileS2*, which confers high-level mupirocin resistance, or the multiresistance gene *cfr*, which confers combined resistance to phenicols, lincosamides, oxazolidinones, pleuromutilins and streptogramin A, have also been detected in *S. aureus* and/or *S. pseudintermedius* [24,25].

Thus, antimicrobial chemotherapy of dogs and cats infected with *S. aureus* and *S. pseudintermedius* requires the determination of the antimicrobial resistance pattern of the causative isolate by an antibiogram prior to the application of antimicrobial agents. A meaningful antibiogram, preferentially conducted by determination of minimal inhibitory concentrations (MICs) of the antimicrobial agents tested, ensures that no antimicrobial agents are used to which the respective isolate already shows resistance in vitro. Moreover, it provides guidance to the veterinarian with regard to the choice of the most suitable antimicrobial agent.

In addition to antimicrobial agents, biocides, such as chlorhexidine, have also been evaluated for their efficacy in the topical treatment of infections caused by multiresistant *S. aureus* and *S. pseudintermedius*, e.g., canine superficial pyoderma [26,27,28]. However, most biocides are not used for therapeutical applications but for disinfection of the environment in hospitals, households and on farms. As such, bacteria present in these settings are also exposed to a selection pressure imposed by disinfectants, which may result in a resistance development towards biocides. In selected cases, combined resistance to antimicrobial agents and biocides has been detected [29]. The resistance to biocides can be intrinsic or acquired, with mycobacteria probably being most (intrinsically) resistant to biocides, followed by Gram-negative bacteria and with Gram-positive bacteria being most susceptible [29]. So far, biocide susceptibility testing has often been performed using different testing methods and protocols, which makes the comparison of the results difficult if not impossible [30]. Recently, a harmonized protocol for biocide susceptibility testing via broth microdilution has been established [30] and hopefully will lead to comparable biocide testing results in the future. There are reports about low-level resistance or reduced susceptibility to quarternary ammonium compounds among staphylococci [31], commonly mediated by *qac* genes [32]. In the present study, the susceptibility to four biocides was determined, including benzalkonium chloride (a quarternary ammonium compound), chlorhexidine and polyhexanide (two biguanides), and octenidine (a bispyridine), which represent commonly used biocide classes.

The aim of the present study was to evaluate *S. aureus* and *S. pseudintermedius* isolates from infections of cats and dogs for their antimicrobial susceptibility to a wide range of antimicrobial agents including the detection of selected resistance genes but also for their susceptibility to four different biocides.

## 2. Results

### 2.1. Origin of the Isolates

In total, 114 isolates, including 62 *S. aureus* (19 from infections of dogs and 43 from infections of cats) and 52 *S. pseudintermedius* (41 from infections of dogs and 11 from infections of cats), from diagnostic submissions to the Institute of Microbiology and Epizootics, Department of Veterinary Medicine, Freie Universität Berlin, Berlin, Germany were included in this study. The majority of the dogs and cats, from which the isolates originated, were presented with a wide range of infections at the Small Animal Clinic, Department of Veterinary Medicine, Freie Universität Berlin, Berlin, Germany and several other veterinary clinics/practices in Germany between 01/2017 and 08/2018. *S. aureus* was most frequently isolated from wounds (*n* = 8 (dogs); *n* = 15 (cats)), skin infections (*n* = 2 (dogs); *n* = 8 (cats)), infections of the upper respiratory tract (*n* = 3 (dogs); *n* = 4 (cats)), infections of the ears (*n* = 0 (dogs); *n* = 5 (cats)), and urinary tract infections (*n* = 0 (dogs); *n* = 4 (cats)). The remaining *S. aureus* isolates originated from bone infections, soft tissue infections, or blood cultures. *S. pseudintermedius* was most frequently isolated from skin infections (*n* = 15 (dogs); *n* = 4 (cats)), wounds (*n* = 11 (dogs); *n* = 0 (cats)), infections of the ears (*n* = 7 (dogs); *n* = 1 (cats)), and urinary tract infections (*n* = 4 (dogs); *n* = 2 (cats)). The remaining *S. pseudintermedius* isolates originated from upper respiratory tract infections, soft tissue infections, or eye infections. All *S. aureus* and *S. pseudintermedius* isolates were from individual unrelated dogs and cats.

### 2.2. Antimicrobial Susceptibility of Feline S. aureus Isolates

The distribution of the MIC data of the feline *S. aureus* isolates is shown in Table 1. Among the 43 *S. aureus* isolates from infections of cats, 33 (76.7%) showed resistance to penicillin G by applying the clinical breakpoint (S ≤ 0.12 mg/L; R ≥ 0.25 mg/L) from CLSI document M100 [33] as no veterinary-specific breakpoints are available for staphylococci from dogs and cats in the CLSI document VET01S [23]. Twenty-six isolates (60.5%) exhibited ampicillin resistance by applying the cat-specific clinical breakpoints [23] with another two isolates being borderline penicillin-resistant (MIC 0.25 mg/L) and ampicillin-intermediate (MIC 0.5 mg/L), as well as five isolates being penicillin-resistant and borderline ampicillin-susceptible (MIC 0.25 mg/L). In total, 14 isolates (32.6%) were resistant to oxacillin with MICs of ≥4 mg/L [23,33]. Twenty-three isolates were classified as resistant to amoxicillin–clavulanic acid [23]. This included all oxacillin-resistant isolates as well as nine penicillin- and ampicillin-resistant isolates. These latter nine isolates were borderline amoxicillin–clavulanic acid resistant with MICs of 1/0.5 mg/L. All β-lactam-resistant *S. aureus* isolates carried at least one β-lactam resistance gene. The distribution of the corresponding resistance genes was as follows: 19 isolates carried the β-lactamase gene *blaZ*, another eight isolates *blaZ* and the methicillin resistance gene *mecA*, while four isolates harbored only *mecA* and two isolates only *mecC*. Phenotypic oxacillin resistance and the presence of the genes *mecA* or *mecC* classified 14 isolates as MRSA. In contrast to the remaining 29 methicillin-susceptible *S. aureus* (MSSA) isolates, the 14 MRSA isolates exhibited higher MICs to cephalothin (1–128 mg/L), cefotaxime (8–≥64 mg/L), cefoperazone (8–≥64 mg/L), and ceftiofur (4–≥128 mg/L).

Only five (9.3%) of the 43 isolates were tetracycline-resistant with MICs ranging between 1 and 128 mg/L. As no cat-specific clinical breakpoints for tetracyclines were available in VET01S [23], the clinical breakpoints for dogs were applied in accordance with the CLSI document VET09 [34]. The two isolates with MICs of 16 and 32 mg/L carried a *tet*(K) gene, the two isolates with MICs of 64 and 128 mg/L harbored *tet*(K) and *tet*(M) genes, while the single isolate with an MIC of 1 mg/L did not harbor one of the *tet* genes tested. All *tet* gene-carrying isolates also exhibited elevated doxycycline MICs (1–8 mg/L). Macrolide resistance was observed in 11 (25.6%) of the 43 feline *S. aureus* isolates. Again, the clinical breakpoints from document M100 for erythromycin as the class representative of macrolides were applied [33]. All macrolide-resistant isolates exhibited erythromycin MICs of 8–≥64 mg/L and also elevated the MICs of tilmicosin (1–≥256 mg/L), tylosin (1–≥256 mg/L), and tulathromycin (8–≥64 mg/L). Solely *erm* genes were detected in these isolates, including *erm*(A) (*n* = 2), *erm*(A) + *erm*(B) (*n* = 3), *erm*(B) (*n* = 1), *erm*(C) (*n* = 4), and *erm*(T) (*n* = 1). Three of these isolates—two carrying *erm*(C) genes and the single isolate with *erm*(T)—showed high MICs for erythromycin (8–≥64 mg/L) but distinctly lower MICs for the 16-membered macrolides tilmicosin (1 or 2 mg/L) and tylosin (1 or 2 mg/L), as well as the lincosamide clindamycin (0.06 or 0.12 mg/L). This is indicative of inducible macrolide/lincosamide resistance as 16-membered macrolides and lincosamides are not inducers while the 14-membered macrolide erythromycin is an inducer of the respective *erm* genes.

Among the tested aminoglycosides, resistance to gentamicin was seen only in a single isolate with a gentamicin MIC of 32 mg/L [23], which was positive for the gene *aacA-aphD*. No CLSI-approved clinical breakpoints are available for streptomycin and neomycin. Two isolates with elevated streptomycin MICs of 256 and ≥1024 mg/L, both carrying the streptomycin resistance gene *aadE*, were detected. In addition, the three isolates with elevated neomycin MICs harbored neomycin resistance genes. The isolate with a neomycin MIC of 16 mg/L carried an *aadD* gene while the two isolates with neomycin MICs of 64 mg/L were positive for the neomycin resistance gene *aphA3*. Fluoroquinolone resistance was seen in ten (23.3%) feline *S. aureus* isolates, all of which ranged in their MICs between 2–≥32 mg/L (enrofloxacin), 8–≥32 mg/L (marbofloxacin), and 8–≥32 mg/L (ciprofloxacin). These ten isolates also had elevated nalidixic acid MICs of 64–≥256 mg/L.

In addition, all but one isolate had tiamulin MIC values in the range between 0.25–1 mg/L. The single isolate exhibited a high tiamulin MIC of ≥128 mg/L, had a high clindamycin MIC of ≥128 mg/L and was borderline resistant to quinupristin-dalfopristin (MIC 4 mg/L). It harbored a *lnu*(B) gene for lincosamide resistance, a *lsa*(E) gene for combined resistance to lincosamides, pleuromutilins and streptogramin A antibiotics (dalfopristin) as well as an *erm*(B) gene for combined resistance to macrolides, lincosamides and streptogramin B antibiotics (quinupristin). Another single isolate was resistant to trimethoprim-sulfamethoxazole (MIC 8/152 mg/L) and harbored the trimethoprim resistance gene *dfrG*. All 43 feline isolates showed unimodal MIC distributions for linezolid (range 0.5–4 mg/L) and vancomycin (range 0.25–2 mg/L) and were classified as susceptible to these antimicrobial agents. Moreover, florfenicol MICs also showed a unimodal distribution (range 2–8 mg/L) (Table 1). Based on the low florfenicol and tiamulin MICs, the respective isolates were tentatively considered susceptible.

In total, 10/43 isolates were pansusceptible, i.e., susceptible to all antimicrobial agents tested, 17 were resistant to only one class, six isolates to two classes, and 10 isolates to at least three classes of antimicrobial agents. These latter isolates (23.3%) were classified as multiresistant [35]. The multiple antimicrobial resistance (MAR) index of the feline *S. aureus* isolates was 0.126.

### 2.3. Antimicrobial Susceptibility of Canine S. aureus Isolates

The distribution of the MIC data of the canine *S. aureus* isolates is shown in Table 2. Among the 19 *S. aureus* isolates from infections of dogs, 17 (89.5%) showed resistance to penicillin G [33]. Fifteen isolates (78.9%) exhibited ampicillin resistance by applying the aforementioned dog-specific clinical breakpoints applicable to *S. pseudintermedius* [23]. Two isolates were borderline penicillin-resistant (MIC 0.25 mg/L) and borderline ampicillin-susceptible (MIC 0.25 mg/L). In total, seven isolates (36.8%) were resistant to oxacillin with MICs of ≥16 mg/L [23]. Twelve isolates were classified as resistant to amoxicillin-clavulanic acid [23]. This included all oxacillin-resistant isolates as well as five penicillin- and ampicillin-resistant isolates. Four of these latter five isolates were borderline amoxicillin–clavulanic acid resistant with MICs of 1/0.5 mg/L. As seen for the feline *S. aureus* isolates, all β-lactam-resistant canine *S. aureus* isolates carried at least one β-lactam resistance gene. The distribution of the corresponding resistance genes was as follows: ten isolates carried the β-lactamase gene *blaZ*, another six isolates *blaZ* and *mecA*, a single isolate only *mecA*, while *mecC* was not detected. Phenotypic oxacillin resistance and the presence of the gene *mecA* classified seven isolates as MRSA. In contrast to the remaining 12 MSSA isolates, the seven MRSA isolates exhibited higher MICs to cephalothin (1–128 mg/L), cefotaxime (8–≥64 mg/L), cefoperazone (8–≥64 mg/L), and ceftiofur (2–≥128 mg/L).

Only four (21.1%) of the 19 isolates were tetracycline-resistant with MICs ranging between 1 and 64 mg/L using dog-specific clinical breakpoints [23]. The single isolate with a MIC of 1 mg/L did not harbor a tetracycline resistance gene, while the two isolates with tetracycline MICs of 32 mg/L harbored a *tet*(K) gene and the one isolate with a MIC of 64 mg/L was positive for the *tet*(M) gene. All *tet* gene-carrying tetracycline-resistant isolates also exhibited elevated doxycycline MICs (2–8 mg/L). Macrolide resistance was observed in six (31.6%) of the 19 canine *S. aureus* isolates [33]. All macrolide-resistant isolates exhibited erythromycin MICs of ≥64 mg/L and harbored the resistances genes *erm*(A) (*n* = 1), *erm*(B) (*n* = 2), *erm*(B) + *erm*(C) (*n* = 1), *erm*(T) (*n* = 1) and *msr*(A) (*n* = 1). Low MICs of the non-inducers were seen in the isolate with the inducibly expressed *erm*(T) gene and in the *msr*(A)-carrying isolate.

Among the tested aminoglycosides, resistance to gentamicin was seen in three *aacA-aphD*-carrying isolates with gentamicin MICs of 32–128 mg/L [23,33]. Three isolates with elevated streptomycin MICs of 128–≥1024 mg/L, carrying either the *aadE* or *str* genes, were detected. In addition, four isolates with elevated neomycin MICs ranging between 8 and ≥128 mg/L were identified. They harbored the neomycin resistance genes *aadD* (*n* = 2) or *aphA3* (*n* = 2). Fluoroquinolone resistance was seen in five (26.3%) canine *S. aureus* isolates, all of which ranged in their MICs between 4–≥32 mg/L (enrofloxacin), 16–≥32 mg/L (marbofloxacin), and 16–≥32 mg/L (ciprofloxacin). These isolates also exhibited elevated nalidixic acid MICs of 64–≥256 mg/L.

All 19 canine *S. aureus* isolates showed unimodal MIC distributions for quinupristin-dalfopristin (range 0.25–1 mg/L), trimethoprim-sulfamethoxazole (range 0.06/1.19–1/19 mg/L), linezolid (range 0.5–4 mg/L), and vancomycin (range 0.5–2 mg/L) and were classified as susceptible to these antimicrobial agents. Moreover, the florfenicol (range 2–8 mg/L) and tiamulin (range 0.12–1 mg/L) MICs also showed a unimodal distribution (Table 2) and the respective isolates were considered susceptible.

Only a single canine *S. aureus* isolate was pansusceptible, eight isolates were resistant to only one class, two isolates to two classes and eight isolates to at least three classes of antimicrobial agents. The percentage of multiresistant canine *S. aureus* was 42.1% [35]. The MAR index of canine *S. aureus* isolates was 0.187.

### 2.4. Antimicrobial Susceptibility of Feline S. pseudintermedius Isolates

The distribution of the MIC data of the feline *S. pseudintermedius* isolates is shown in Table 3. Of the 11 *S. pseudintermedius* isolates from infections of cats, ten (90.9%) isolates showed resistance to penicillin G [33]. Among them, seven were also classified as ampicillin-resistant (63.6%) by applying the cat-specific clinical breakpoints for *Staphylococcus* spp. [23]. One penicillin-resistant isolate was classified as ampicillin-intermediate (MIC 0.5 mg/L) while two penicillin-resistant isolates were borderline ampicillin-susceptible (MIC 0.25 mg/L). In total, three isolates (27.3%) were resistant to oxacillin with MICs of 4–≥16 mg/L [23]. These three isolates were also classified as resistant to amoxicillin-clavulanic acid [23]. All β-lactam-resistant feline *S. pseudintermedius* isolates harbored at least one β-lactam resistance gene with eight isolates carrying the β-lactamase gene *blaZ* and three isolates *blaZ* and *mecA*. Phenotypic oxacillin resistance and the presence of the gene *mecA* classified three isolates as MRSP. In contrast to the remaining eight methicillin-susceptible *S. pseudintermedius* (MSSP) isolates, the three MRSP isolates exhibited higher MICs to cephalothin (0.5–64 mg/L), cefotaxime (4–≥64 mg/L), cefoperazone (2–≥64 mg/L), and ceftiofur (4–≥128 mg/L).

Four (36.4%) of the 11 isolates were tetracycline-resistant with MICs ranging between 32 and 64 mg/L using the dog-specific clinical breakpoints due to the lack of cat-specific breakpoints [23]. All four isolates were also doxycycline-resistant (MICs 4–8 mg/L) and carried a *tet*(M) gene. Macrolide resistance was seen in six (54.5%) of the 11 feline *S. pseudintermedius* isolates [33]. All macrolide-resistant isolates exhibited erythromycin MICs of ≥64 mg/L and harbored the resistances genes *erm*(B) (*n* = 4) or *erm*(B) + *erm*(C) (*n* = 2). All six isolates also exhibited high MICs to tilmicosin (≥256 mg/L), tylosin (≥256 mg/L), and clindamycin (4–≥128 mg/L), suggesting that the detected *erm* genes are expressed constitutively.

Resistance to gentamicin was seen in two *aacA-aphD*-carrying isolates (18.2%) with gentamicin MICs of 16 or 32 mg/L [23]. Six isolates (54.5%) with elevated streptomycin MICs of 64–≥1024 mg/L, carrying the *aadE* gene, were identified. The same six isolates also showed elevated neomycin MICs of 8 or 16 mg/L and harbored the resistance gene *aphA3*. Fluoroquinolone resistance was detected in two (18.2%) feline *S. pseudintermedius* isolates with MICs of 16 and ≥32 mg/L (enrofloxacin), 16 and ≥32 mg/L (marbofloxacin), and ≥32 mg/L (ciprofloxacin). These isolates also exhibited elevated nalidixic acid MICs of 128 mg/L.

In addition, the two gentamicin-resistant isolates (18.2%) were also classified as resistant to trimethoprim-sulfamethoxazole (MICs 8/152 or 16/304 mg/L) and harbored the trimethoprim resistance gene *dfrG*. All 11 feline *S. pseudintermedius* isolates showed unimodal MIC distributions for quinupristin-dalfopristin (range 0.12–0.5 mg/L), linezolid (range 0.5–1 mg/L), and vancomycin (range 1–2 mg/L) and were classified as susceptible to these antimicrobial agents. In addition, the florfenicol (range 2–4 mg/L) and tiamulin (range 0.06–0.25 mg/L) MICs also showed a unimodal distribution (Table 3) and the respective isolates were considered as susceptible.

Only a single feline *S. pseudintermedius* isolate was pansusceptible, two isolates were resistant to only one class, two isolates to two classes and six isolates to at least three classes of antimicrobial agents. The percentage of multiresistant feline *S. pseudintermedius* was 54.5% [35]. The MAR index of feline *S. pseudintermedius* isolates was 0.286.

### 2.5. Antimicrobial Susceptibility of Canine S. pseudintermedius Isolates

The distribution of the MIC data of the canine *S. pseudintermedius* isolates is shown in Table 4. Thirty-five (85.4%) of the 41 *S. pseudintermedius* isolates from the infections of dogs exhibited resistance to penicillin G [33]. Among them, 31 were also classified as ampicillin-resistant (75.6%) [23]. The remaining four penicillin G-resistant isolates were classified as borderline ampicillin-susceptible (MIC 0.12–0.25 mg/L). Five isolates (12.2%) were resistant to oxacillin with MICs of 8–≥16 mg/L [23]. These five isolates were also classified as resistant to amoxicillin-clavulanic acid [23]. All β-lactam-resistant canine *S. pseudintermedius* isolates harbored at least one β-lactam resistance gene. Among them, 30 isolates carried the β-lactamase gene *blaZ*, four isolates *blaZ* and *mecA*, while one isolate was only positive for *mecA*. In contrast to the remaining 36 MSSP isolates, the five MRSP isolates exhibited higher MICs to cephalothin (0.5–64 mg/L), cefotaxime (8–≥64 mg/L), cefoperazone (4–≥64 mg/L), and ceftiofur (4–≥128 mg/L).

Ten (24.4%) of the 41 isolates were tetracycline-resistant with MICs ranging between 32 and 64 mg/L [23]. The dog-specific clinical breakpoints applicable to *S. pseudintermedius* also classified all ten isolates as doxycycline-resistant (MICs 4–8 mg/L). These isolates carried a *tet*(M) (*n* = 9) or a *tet*(K) (*n* = 1) gene. Macrolide resistance was seen in eight (19.5%) of the 41 canine *S. pseudintermedius* isolates [33]. All macrolide-resistant isolates exhibited erythromycin MICs of ≥64 mg/L and most of them also elevated MICs of tilmicosin and tylosin. They harbored the resistance genes *erm*(B) (*n* = 5) or *erm*(B) + *erm*(C) (*n* = 3). Two isolates showed inducible macrolide/lincosamide resistance whereas the remaining six isolates exhibited a constitutive resistance phenotype.

The resistance gene *aacA-aphD* was detected in two intermediate and two resistant isolates that varied in their gentamicin MICs between 8 and 32 mg/L [23,33]. Eight isolates (19.5%) with elevated streptomycin MICs of 128–≥1024 mg/L carried the gene *aadE*. The same eight isolates also showed elevated neomycin MICs of 8 or 16 mg/L and harbored the resistance gene *aphA3*. Fluoroquinolone resistance was detected in seven (17.1%) canine *S. pseudintermedius* isolates, all of which ranged in their MICs between 8–≥32 mg/L (enrofloxacin), 16–≥32 mg/L (marbofloxacin), and 8–≥32 mg/L (ciprofloxacin). These isolates also showed elevated nalidixic acid MICs of 64–≥256 mg/L.

In addition, three isolates (7.3%) were classified as resistant to trimethoprim-sulfamethoxazole (MICs 8/152 or 16/304 mg/L) and carried the *dfrG* gene. All 41 canine *S. pseudintermedius* isolates showed unimodal MIC distributions for quinupristin-dalfopristin (range 0.12–0.5 mg/L), linezolid (range 0.5–2 mg/L), and vancomycin (range 0.5–2 mg/L) and were classified as susceptible to these antimicrobial agents. In addition, the florfenicol (range 2–4 mg/L) and tiamulin (range 0.06–0.5 mg/L) MICs also showed unimodal distributions (Table 4) and the corresponding isolates were also considered susceptible.

Five canine *S. pseudintermedius* isolates were pansusceptible, 21 isolates were resistant to only one class, five isolates to two classes, and ten isolates to at least three classes of antimicrobial agents. The percentage of multiresistant canine *S. pseudintermedius* was 24.4% [35]. The MAR index of canine *S. pseudintermedius* isolates was 0.155.

### 2.6. Biocide Susceptibility of Feline and Canine S. aureus and S. pseudintermedius Isolates

**Figure 1:**
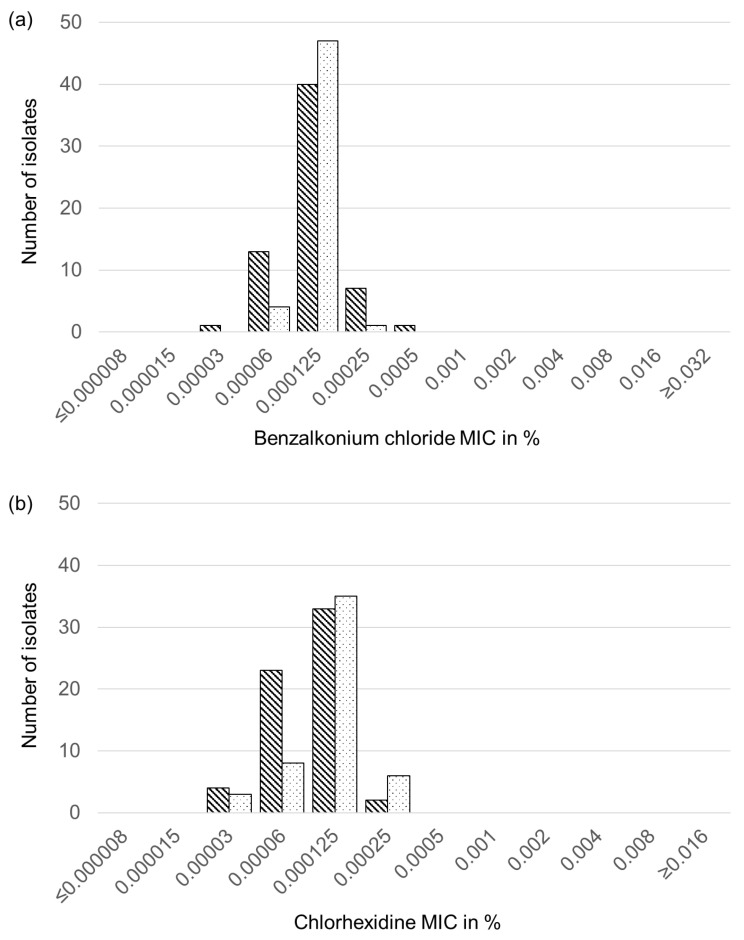

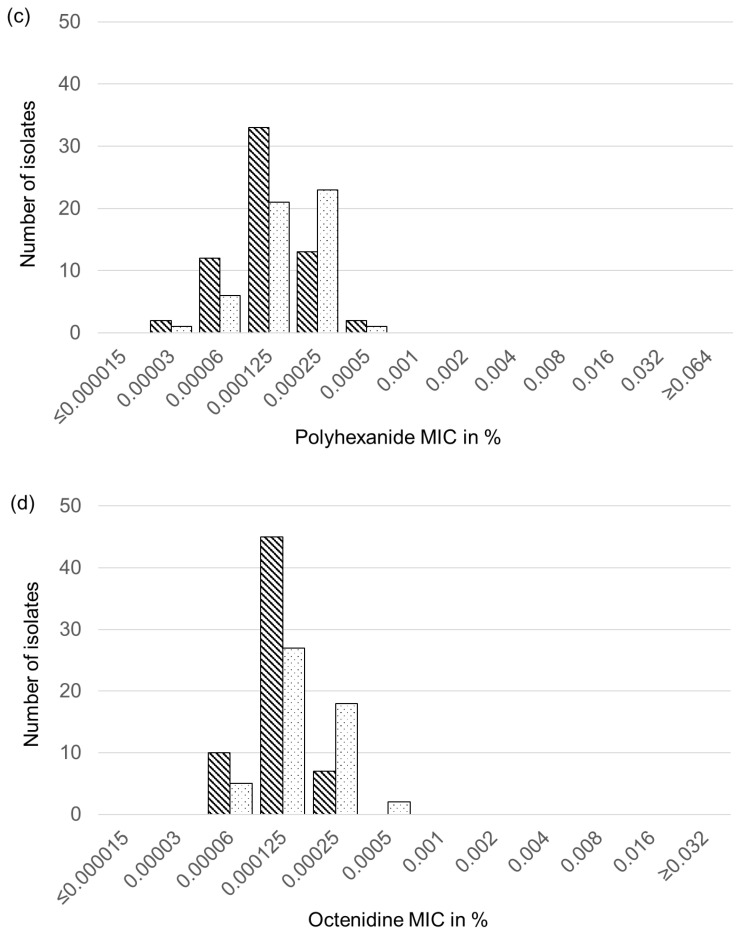
Distributions of the MIC values of the 62 feline and canine *S. aureus* isolates (striped columns) and the 52 feline and canine *S. pseudintermedius* isolates (dotted columns) for the four biocides tested: (**a**) benzalkonium chloride, (**b**) chlorhexidine, (**c**) polyhexanide, and (**d**) octenidine.

Analysis of the 43 feline and 19 canine *S. aureus* isolates as well as the 11 feline and 41 canine *S. pseudintermedius* isolates for their susceptibility to the four different biocides benzalkonium chloride, chlorhexidine, polyhexanide, and octenidine yielded unimodal distributions of the MICs for each biocide. No striking differences in the MICs for the respective biocides were seen between the canine and feline isolates of each of the two staphylococcal species. Moreover, the biocide-specific MIC distributions were also very similar for the *S. aureus* and *S. pseudintermedius* isolates (Figure 1).

For benzalkonium chloride, the majority of the *S. aureus* isolates (*n* = 60) exhibited MICs of 0.00006–0.00025% with only single isolates showing lower (0.00003%) or higher (0.005%) values (Figure 1a). The corresponding MIC values of all 52 *S. pseudintermedius* isolates ranged between 0.00006–0.00025%. For chlorhexidine and polyhexanide, the MIC values of all *S. aureus* and *S. pseudintermedius* isolates were in the range between 0.00003 and 0.0025% (Figure 1b,c). The MIC values for octenidine of all *S. aureus* and all but two *S. pseudintermedius* isolates varied between 0.00003 and 0.00025%. The remaining two *S. pseudintermedius* had a one-dilution step higher MIC of 0.0005% (Figure 1d).

## 3. Discussion

Antimicrobial susceptibility testing in routine diagnostics is commonly intended to guide antimicrobial chemotherapy. If bacteria from animals are tested, veterinary-specific clinical breakpoints need to be applied to categorize bacteria as resistant, susceptible, or intermediate (if this category is available) to a specific antimicrobial agent or combination of antimicrobial agents [35,36]. For *S. aureus* and *S. pseudintermedius* isolates from dogs and cats, veterinary-specific clinical breakpoints for numerous antimicrobial agents are listed in the CLSI document VET01S [23]. However, dog- or cat-specific clinical breakpoints are still missing for important antimicrobial agents, such as penicillin G, oxacillin, gentamicin, trimethoprim-sulfamethoxazole, linezolid, vancomycin, and quinupristin-dalfopristin. It should be noted that the latter three agents are not approved for use in dogs and cats worldwide but may be used in single cases if no appropriate antimicrobial agent approved for veterinary use is available. Oxacillin is not used therapeutically; instead, the determination of oxacillin resistance is of diagnostic value to identify MRSA or MRSP isolates. The same is true for nalidixic acid as high nalidixic acid MICs point towards a resistance development against fluoroquinolones. As the next best approximation, human-specific clinical breakpoints from the CLSI document M100 are applied [33]. The classification of canine and feline *S. aureus* and *S. pseudintermedius* isolates based on the use of these clinical breakpoints proved to be in agreement with the detection of resistance genes as shown, e.g., for penicillin G, oxacillin, and gentamicin in this study. In the case of tetracycline, the dog-specific clinical breakpoints (S ≤ 0.25, I 0.5, R ≥ 1 mg/L) from document VET01S are lower than the human-specific clinical breakpoints (S ≤ 4, I 8, R ≥ 16 mg/L). The human breakpoints, however, better reflect the presence of tetracycline resistance genes. For other antimicrobial agents tested, such as tiamulin, tilmicosin or florfenicol, only veterinary-specific clinical breakpoints for non-staphylococcal bacteria from food-producing animals (pigs and/or cattle) are currently available. The use of such clinical breakpoints for the categorization of staphylococcal isolates must be done with caution [34]. Even though no veterinary-specific breakpoints applicable to staphylococci are available, isolates with the observed low florfenicol and tiamulin MICs were tentatively considered as susceptible in accordance with previously published studies in which phenicol-resistant and/or pleuromutilin-resistant isolates showed distinctly higher MICs and carried specific phenicol and/or pleuromutilin resistance genes [37,38].

The calculation of the MAR index must also be done with caution as bias can be introduced when including multiple antimicrobial agents of the same class, e.g., ciprofloxacin, enrofloxacin and marbofloxacin, in the calculation. Usually resistance genes or resistance-mediating mutations confer resistance to several if not all members of a certain class of antimicrobial agents. To avoid such bias, we included only one substance per class, usually the class representative, in our calculation. As no class representative can be defined for aminoglycosides, we included all three aminoglycosides tested. The MAR indices observed in this study were similar for feline (0.126) and canine *S. aureus* (0.187) as well as canine *S. pseudintermedius* (0.155) isolates, while the MAR index of feline *S. pseudintermedius* isolates was distinctly higher at 0.286. However, feline *S. pseudintermedius* isolates represented the smallest group comprising only 11 isolates. In small test populations of, e.g., 10–20 isolates, few multiresistant isolates will have a disproportionately high influence on the MAR index. In the present study, six of the 11 isolates showed a multiresistance phenotype. Thus, higher MAR indices obtained from small test populations must not be overestimated.

There is only one other study from Germany that can be used for the comparison of the resistance rate. This study was conducted in 2004–2006 and summarized *S. aureus* and members of the *Staphylococcus intermedius* group, including *S. pseudintermedius*, as coagulase-positive staphylococci. In this study, independent isolates from different infections of dogs and cats were collected and tested for their susceptibility to a variety of antimicrobial agents by broth microdilution according to CLSI recommendations [39]. Penicillin G resistance was the dominant resistance property in the BfT-GermVet study with resistance rates of 53.5% recorded for 101 coagulase-positive staphylococci from infections of the skin/ear/mouth of dogs and cats but also of 66.7% recorded for 57 coagulase-positive staphylococci from respiratory tract infections of dogs and cats [39]. In the present study, the susceptibility data also showed that penicillin G resistance was the dominant resistance property among all four groups of isolates tested, but the resistance rate for the entire test collection was distinctly higher at 83.3% (95/114). In addition, the numbers of oxacillin-resistant isolates varied distinctly between the two studies. While oxacillin resistance was rarely detected in the BfT-GermVet study, i.e., 1.0% (1/101) and 1.8% (1/57) in the two test populations, more than one quarter of the isolates in the current study (25.4%, 29/114) showed oxacillin resistance.

Macrolide resistance (31/114, 27.2%) was the second most prevalent resistance property in the present study. This resistance rate corresponded closely to that of the coagulase-positive staphylococci from infections of skin/ear/mouth (27/101, 26.7%) and that of respiratory tract infections (13/57, 22.8%) of dogs and cats in the BfT-GermVet study [34]. Tetracycline resistance was the second most prevalent resistance property in the BfT-Germ-Vet study with resistance rates of 36.6% (37/101) and 35.1% (20/57) for the two test populations [39]. In the present study, however, tetracycline was seen only in 19.3% (22/114) of the isolates. In contrast, fluoroquinolone resistance was detected in 21.1% (24/114) of the isolates of the present study whereas the BfT-GermVet isolates exhibited fluoroquinolone resistance at distinctly lower frequencies of 2.0% (2/101) and 3.5% (2/57) [39]. Resistances to the remaining antimicrobial classes—if available at all—were below 10% in both studies.

Whether the higher penicillin G and also fluoroquinolone resistance rates in the present study are due to an increased use of β-lactams and fluoroquinolones in small animal medicine during recent years remains to be answered. Currently, no specific consumption figures of antimicrobial agents applicable to dogs and cats from Germany are available. However, most isolates of the present study originated from cats and dogs admitted to a referral veterinary clinic and likely had been pre-treated before admission to these clinics. This might explain the higher percentages of β-lactam (including methicillin) and fluoroquinolone resistance.

The resistance genes detected in the present study corresponded well to those identified in previous studies. As such, the β-lactam resistance genes *blaZ*, *mecA* and to a lesser extent *mecC* have been identified in *S. aureus* [9,40] as well as *blaZ* and *mecA* in *S. pseudintermedius* from dogs and cats [12,17,18]. The tetracycline resistance genes *tet*(K) and *tet*(M) have been identified as the dominant *tet* genes in *S. aureus* and *S. pseudintermedius* from animals, including dogs and cats [9,12,17,18]. The macrolide resistance genes *erm*(A), *erm*(B), *erm*(C) and *msr*(A)—alone or in various combinations—are known to occur in *S. aureus* and *S. pseudintermedius* [9,12,17,18]. However, the presence of the gene *erm*(T) in *S. aureus* and *S. pseudintermedius* isolates from dogs and cats was a novel observation. The gene *aacA-aphD* (also known as *aac(6′)-Ie–aph(2′)-Ia*) is the most frequently encountered gentamicin resistance gene among staphylococci [9,12,17,18]. In addition, the streptomycin resistance genes *aadE* (also known as *ant(6′)-Ia*) and *str* are common streptomycin resistance genes in *S. aureus* while *aadE* is more prevalent in *S. pseudintermedius* [9,12,17,18]. A similar situation is true for the kanamycin/neomycin resistance genes *aadD* and *aphA3*, with the latter being more often found in *S. pseudintermedius* [9,12,17,18]. The simultaneous presence of the aminoglycoside resistance genes *aadE* and *aphA3* in *S. pseudintermedius* is most likely due to a Tn*5405*-like element on which these resistance genes reside. Such elements have been described to occur preferentially in canine and feline *S. pseudintermedius* isolates [12,17,18,41].

The biocide MICs determined in the present study showed unimodal distributions that mainly included three dilution steps, regardless of the biocide and the staphylococcal species tested. As such, no hints towards isolates with a reduced biocide susceptibility were found. Moreover, the highest MIC value for benzalkonium chloride measured was 0.0005%, which is far below the concentration used for disinfection of the skin of 0.01–0.2% [42]. This was in accordance with previous studies investigating 19 *S. aureus* isolates from horses [43] and 17 isolates from beavers [44]. In comparison, a study on *S. aureus* from primates identified some isolates with a slightly elevated MICs of 0.0004%, which harbored the *qacC* gene [45]. Currently, three different shampoos that contain chlorhexidine (in combination with miconazole) are approved for use in dogs and cats in Germany. Again, the highest chlorhexidine MIC measured in this study (0.00025%) was far below the concentration of up to 4% in shampoos for veterinary applications [26,27]. Moreover, these MICs were in accordance with the results obtained by testing the isolates from horses [43] and beavers [44] No polyhexanide- or octenidine-containing formulations are currently approved for veterinary use. The MICs for polyhexidine and octenidine were also in a similar range to those determined for the *S. aureus* isolates from beavers, with MICs for polyhexanide of 0.000125–0.001% and for octenidine of 0.00006–0.00025% [44].

## 4. Materials and Methods

### 4.1. Identification of S. aureus and S. pseudintermedius

*S. aureus* and *S. pseudintermedius* were isolated from the respective swabs by previously described standard procedures [46]. In brief, the swabs were streaked directly on Columbia agar containing 5.0% (*v*/*v*) sheep blood (bioMérieux, Nürtingen, Germany) and incubated at 37 °C for 18 h. In cases of low bacterial growth after 18 h, the plates were further incubated and re-inspected after 36 h. Bacterial isolates were presumably identified as coagulase-positive *Staphylococcus* spp. based on colony morphology, Gram-stain appearance, a positive catalase reaction, a positive tube coagulase test and hemolysis patterns. Species assignment of *S. aureus* and *S. pseudintermedius* was verified by matrix-assisted laser desorption ionization-time of flight mass spectrometry (MALDI-TOF MS) (Bruker Daltonik, Bremen, Germany) [46,47].

### 4.2. Antimicrobial Susceptibility Testing and Detection of Resistance Genes

Minimal inhibitory concentrations (MICs) of the 114 *S. aureus* and *S. pseudintermedius* isolates were determined for 24 antimicrobial agents and three combinations of antimicrobial agents by broth microdilution according to the recommendations of the CLSI [23,33]. For this, the microtitre plate layouts (Sensititre^®^, Thermo Fisher Scientific, Waltham, MA, USA) used in the national resistance monitoring program GE*RM*-Vet, were also used in this study. The antimicrobial agents tested and the test ranges in mg/L were as follows: amoxicillin-clavulanic acid (0.03/0.015–64/32), ampicillin (0.03–64), cefoperazone (0.06–32), cefotaxime (0.015–32), ceftiofur (0.03–64), cephalothin (0.06–128), ciprofloxacin (0.008–16), clindamycin (0.03–64), doxycycline (0.06–128), enrofloxacin (0.008–16), erythromycin (0.015–32), florfenicol (0.12–256), gentamicin (0.12–256), linezolid (0.03–64), marbofloxacin (0.008–16), nalidixic acid (0.06–128), neomycin (0.12–64), oxacillin (0.015–8), penicillin G (0.015–32), quinupristin-dalfopristin (0.015–32), streptomycin (0.25–512), tetracycline (0.12–256), tiamulin (0.03–64), tilmicosin (0.06–128), trimethoprim-sulfamethoxazole (0.015/0.3–32/608), tylosin (0.06–128), and vancomycin (0.015–32). *S. aureus* ATCC^®^ (Manassas, VA, USA) 29213 served as a quality control strain.

Cat-specific clinical breakpoints, listed in the CLSI document VET01S [23], were applied to ampicillin, amoxicillin–clavulanic acid, enrofloxacin and marbofloxacin (all applicable to *Staphylococcus* spp.) for all feline isolates, but also for canine isolates when no dog-specific breakpoints were available. Dog-specific clinical breakpoints [23] were applied to amoxicillin–clavulanic acid, clindamycin, enrofloxacin, marbofloxacin, and tetracycline (applicable to *Staphylococcus* spp.) as well as cephalothin (applicable to *S. aureus* and *S. pseudintermedius*) for all canine isolates, but also for feline isolates when no cat-specific breakpoints were available. In the case of ampicillin, where dog-specific breakpoints only applicable to *S. pseudintermedius* were listed in the CLSI document VET01S [23], we also used these breakpoints for the classification of the canine *S. aureus* isolates. The dog-specific doxycycline breakpoints for *S. pseudintermedius* were utilized for the classification of canine and feline *S. aureus*, as well as for feline *S. pseudintermedius*. All these extrapolations are in agreement with the recommendations given in the CLSI document VET09 [34].

In the absence of veterinary-specific clinical breakpoints for staphylococci from dogs and cats in the CLSI document VET01S [23], human-specific clinical breakpoints from CLSI document M100 [33] were applied for ciprofloxacin, erythromycin, gentamicin, linezolid, penicillin G, oxacillin, trimethoprim-sulfamethoxazole, and vancomycin. The quinupristin-dalfopristin breakpoints applicable to human *S. aureus* isolates [33] served for the classification of feline and canine *S. aureus* and *S. pseudintermedius* isolates. Clinical breakpoints for cefoperazone and *S. aureus* are only applicable to isolates from bovine mastitis. No clinical breakpoints applicable to staphylococci are currently available for cefotaxime, ceftiofur, florfenicol, nalidixic acid, neomycin, streptomycin, tiamulin, tilmicosin, or tylosin.

Antimicrobial resistance genes were detected by specific PCR assays as described previously for β-lactam resistance genes [48,49,50], tetracycline resistance genes [51,52], macrolide/lincosamide resistance genes [38,53,54], aminoglycoside resistance genes [55], and trimethoprim resistance genes [38,56,57]. All PCR experiments were performed on a T3000 Trio thermocycler (Biometra, Göttingen, Germany). Appropriate reference strains from the strain collection of the Institute of Microbiology and Epizootics, Department of Veterinary Medicine, Freie Universität Berlin, Berlin, Germany that carried the respective resistance genes were used as positive controls and purified water as negative controls in the PCR assays.

### 4.3. Calculation of the MAR Index

The calculation of the multiple antimicrobial resistance (MAR) index followed the proposal of Krumperman [58] using the formula *a*/*(b* × *c)*, with *a* being the sum of all resistance properties observed in the isolates of a specific category, e.g., feline *S. aureus*, *b* is the number of antimicrobial agents tested, and *c* is the number of all isolates in a defined category. In the calculation of the MAR index, bias may be introduced by including several antimicrobial agents from the same class to which an isolate, which carries a specific resistance gene, confers resistance. To avoid such bias, only the class representatives or individual antimicrobial agents, for which no cross-resistance is known, were included in this calculation, i.e., penicillin G, erythromycin, clindamycin, gentamicin, streptomycin, neomycin, enrofloxacin, tetracycline, trimethoprim-sulfamethoxazole, florfenicol, linezolid, quinupristin-dalfopristin, tiamulin, and vancomycin. Even if there were no clinical breakpoints for neomycin, streptomycin, and tiamulin applicable to feline and canine *S. aureus* and *S. pseudintermedius*, isolates that showed high MIC values in a bimodal MIC distribution and carried a respective resistance gene were considered resistant.

### 4.4. Biocide Susceptibility Testing

Biocide susceptibility testing for benzalkonium chloride, chlorhexidine, octenidine dihydrochloride (octenidine), and polyhexamethylene biguanide hydrochloride (polyhexanide) was performed by broth microdilution using commercial microtitre plates (sifin diagnostics GmbH, Berlin, Germany) which contained the biocides to be tested in 11 or 12 two-fold dilution steps, i.e., benzalkonium chloride (0.000008–0.016%), chlorhexidine (0.000008–0.008%), polyhexanide (0.000016–0.032%), and octenidine (0.000016–0.016%). Performance of biocide susceptibility followed a previously published protocol [30] modified as follows. The inoculum was prepared by adding 30 µL bacterial suspension of a density of 0.5 McFarland to 12 mL of single-concentrated tryptic soy broth (TSB) and the microtitre plates were inoculated with 100 µL per well according to the manufacturer’s recommendation.

## 5. Conclusions

The results of this study showed that *S. aureus* and *S. pseudintermedius* play a role in a wide variety of infections of cats and dogs. A considerable number of feline and canine *S. aureus* and *S. pseudintermedius* isolates were (multi-) resistant to antimicrobial agents commonly used in small animal medicine. In particular, the high numbers of methicillin- and or fluoroquinolone-resistant isolates demand the performance of antimicrobial susceptibility testing of the causative staphylococci before treating cats and dogs with antimicrobial agents. The results of a meaningful antibiogram—as performed and evaluated *lege artis*—will aid the veterinarian in the choice of the most efficient antimicrobial agents and prevent the application of antimicrobial agents to which the *S. aureus* or *S. pseudintermedius* isolates already display resistance under laboratory conditions. The considerable increase in penicillin-, methicillin- and fluoroquinolone-resistant staphylococci isolated from a comparable companion animal population within roughly a decade highlights the urgent need of the rational use of antimicrobial agents—especially of those that are critically important for human medicine. In contrast to the results of antimicrobial susceptibility testing, the results of biocide susceptibility testing did not point towards the development of resistance to any of the four biocides tested among the *S. aureus* and *S. pseudintermedius* isolates. The measured biocide minimal inhibitory concentrations were distinctly below the corresponding in-use concentrations, which may suggest good efficacy.

## Figures and Tables

**Table 1:** Minimal inhibitory concentrations of the 43 feline *S. aureus* isolates.

Antimicrobial Agent(s)	Number of Isolates for Which the MIC Value (mg/L) Is																	
	0.008	0.015	0.03	0.06	0.12	0.25	0.5	1	2	4	8	16	32	64	128	256	512	1024
Penicillin G		5	5	-	-	5	1	3	4	6	3	5	3	3				
Ampicillin				6	3	6	2	2	7	5	4	3	5	-	-			
Amoxicillin-clavulanic acid (2:1)			1	1	5	11	2	8	3	4	-	6	2	-	-			
Oxacillin		-	-	4	2	8	12	3	-	-	1	13						
Cephalothin				-	6	13	10	3	2	1	1	4	2	-	1	-		
Cefotaxime		-	-	-	-	1	2	1	12	13	3	2	1	8				
Cefoperazone				-	-	-	3	2	15	9	4	2	-	8				
Ceftiofur			-	-	-	3	-	20	6	5	1	-	1	3	4			
Florfenicol					-	-	-	-	19	21	3	-	-	-	-	-	-	
Erythromycin		-	-	-	1	17	11	2	-	-	1	-	-	10				
Tylosin				-	-	-	1	10	21	3	-	-	-	-	-	8		
Tilmicosin				-	-	-	1	15	18	1	-	-	-	-	-	8		
Clindamycin			-	7	22	5	-	-	-	-	-	-	-	1	8			
Streptomycin						-	-	-	1	4	24	11	1	-	-	1	-	1
Gentamicin					2	19	16	5	-	-	-	-	1	-	-	-	-	
Neomycin					4	14	17	5	-	-	-	1	-	2	-			
Ciprofloxacin	-	-	-	-	3	11	16	2	1	-	1	3	6					
Enrofloxacin	-	-	-	2	18	10	1	2	1	3	1	-	5					
Marbofloxacin	-	-	-	-	-	12	19	2	-	-	1	4	5					
Nalidixic acid				-	-	-	-	-	-	-	-	7	13	11	5	7		
Trimethoprim-sulfamethoxazole (1:19)		-	-	30	7	1	2	1	1	-	1	-	-	-				
Tetracycline					17	20	1	1	-	-	-	1	1	1	1	-	-	
Doxycycline				12	23	2	1	2	1	-	2	-	-	-	-	-		
Linezolid			-	-	-	-	6	28	7	2	-	-	-	-	-			
Tiamulin			-	-	-	7	18	17	-	-	-	-	-	-	1			
Vancomycin		-	-	-	-	1	9	32	1	-	-	-	-	-				
Quinupristin-dalfopristin		-	-	-	2	12	24	4	-	1	-	-						

The black areas are the test ranges not included in the test panels for the respective antimicrobial agents. Isolates that had no growth in any of the concentrations were given the lowest MIC value. Isolates with growth in all tested concentrations were given the next serially higher MIC value above the highest tested concentration (white number on black background). The MIC values of amoxicillin-clavulanic acid (2:1) and trimethoprim-sulfamethoxazole (1:19) are expressed as the MIC values of amoxicillin and trimethoprim, respectively. Different gray shading indicates the categories: susceptible—light gray; intermediate—middle gray; resistant—dark gray. For the clinical breakpoints used, please see Materials and Methods, Section 4.2.

**Table 2:** Minimal inhibitory concentrations of the 19 canine *S. aureus* isolates.

Antimicrobial Agent (s)	Number of Isolates for Which the MIC Value (mg/L) Is																	
	0.008	0.015	0.03	0.06	0.12	0.25	0.5	1	2	4	8	16	32	64	128	256	512	1024
Penicillin G		-	2	-	-	2	1	1	-	-	4	5	4	-				
Ampicillin			-	-	2	2	2	2	1	-	4	2	4	-	-			
Amoxicillin-clavulanic acid (2:1)			-	-	-	7	-	4	1	1	1	3	2	-	-			
Oxacillin		-	-	-	2	5	4	1	-	-	-	7						
Cephalothin				-	2	6	4	1	1	1	1	-	1	-	2	-		
Cefotaxime		-	-	-	-	-	2	-	5	5	1	1	1	4				
Cefoperazone				-	-	-	2	-	7	3	2	-	-	5				
Ceftiofur			-	-	-	2	-	6	5	1	-	1	-	2	2			
Florfenicol					-	-	-	-	9	8	2	-	-	-	-	-	-	
Erythromycin		-	-	-	-	6	7	-	-	-	-	-	-	6				
Tylosin				-	-	-	-	2	13	-	-	1	-	-	-	3		
Tilmicosin				-	-	-	-	3	11	1	1	-	-	-	-	3		
Clindamycin			1	2	11	1	-	-	-	-	1	1	-	-	2			
Streptomycin						-	-	-	-	1	9	6	-	-	1	1	-	1
Gentamicin					2	9	4	1	-	-	-	-	2	-	1	-	-	
Neomycin					-	7	6	2	-	-	1	2	-	-	1			
Ciprofloxacin	-	-	-	-	1	6	7	-	-	-	-	1	4					
Enrofloxacin	-	-	-	-	13	1	-	-	-	2	1	1	1					
Marbofloxacin	-	-	-	-	-	7	7	-	-	-	-	1	4					
Nalidixic acid				-	-	-	-	-	-	-	-	2	11	2	1	3		
Trimethoprim-sulfamethoxazole (1:19)		-	-	15	2	1	-	1	-	-	-	-	-	-				
Tetracycline					10	5	-	1	-	-	-	-	2	1	-	-	-	
Doxycycline				10	5	-	1	-	2	-	1	-	-	-	-	-		
Linezolid			-	-	-	-	3	14	1	1	-	-	-	-	-			
Tiamulin			-	-	3	1	7	8	-	-	-	-	-	-	-			
Vancomycin		-	-	-	-	-	1	16	2	-	-	-	-	-				
Quinupristin-dalfopristin		-	-	-	-	4	14	1	-	-	-	-						

The black areas are the test ranges not included in the test panels for the respective antimicrobial agents. Isolates that had no growth in any of the concentrations were given the lowest MIC value. Isolates with growth in all tested concentrations were given the next serially higher MIC value above the highest tested concentration (white number on black background). The MIC values of amoxicillin-clavulanic acid (2:1) and trimethoprim-sulfamethoxazole (1:19) are expressed as the MIC values of amoxicillin and trimethoprim, respectively. Different gray shading indicates the categories: susceptible—light gray; intermediate—middle gray; resistant—dark gray. For the clinical breakpoints used, please see Materials and Methods, Section 4.2.

**Table 3:** Minimal inhibitory concentrations of the 11 feline *S. pseudintermedius* isolates.

Antimicrobial Agent (s)	Number of Isolates for Which the MIC Value (mg/L) Is																	
	0.008	0.015	0.03	0.06	0.12	0.25	0.5	1	2	4	8	16	32	64	128	256	512	1024
Penicillin G		1	-	-	-	-	1	-	2	1	2	1	-	3				
Ampicillin			-	1	-	2	1	3	1	-	-	1	-	2	-			
Amoxicillin-clavulanic acid (2:1)			-	-	2	6	-	1	-	-	1	-	1	-	-			
Oxacillin		-	-	1	2	5	-	-	-	1	-	2						
Cephalothin				1	6	1	1	-	-	-	1	-	-	1	-	-		
Cefotaxime		-	-	-	-	4	4	-	-	1	-	-	-	2				
Cefoperazone				-	-	-	7	1	1	-	1	-	-	1				
Ceftiofur			-	-	-	6	2	-	-	1	-	-	-	1	1			
Florfenicol					-	-	-	-	7	4	-	-	-	-	-	-	-	
Erythromycin		-	-	-	-	5	-	-	-	-	-	-	-	6				
Tylosin				-	-	-	4	1	-	-	-	-	-	-	-	6		
Tilmicosin				-	-	-	1	4	-	-	-	-	-	-	-	6		
Clindamycin			-	-	5	-	-	-	-	1	-	-	1	1	3			
Streptomycin						-	-	-	1	4	-	-	-	1	2	1	-	2
Gentamicin					7	2	-	-	-	-	-	1	1	-	-	-	-	
Neomycin					4	1	-	-	-	-	4	2	-	-	-			
Ciprofloxacin	-	-	-	2	4	3	-	-	-	-	-	-	2					
Enrofloxacin	-	-	-	3	6	-	-	-	-	-	-	1	1					
Marbofloxacin	-	-	-	-	1	7	1	-	-	-	-	1	1					
Nalidixic acid				-	-	-	-	-	-	-	-	3	6	-	2	-		
Trimethoprim-sulfamethoxazole (1:19)		-	-	-	6	-	3	-	-	-	1	1	-	-				
Tetracycline					5	2	-	-	-	-	-	-	2	2	-	-	-	
Doxycycline				6	1	-	-	-	-	2	2	-	-	-	-	-		
Linezolid			-	-	-	-	1	10	-	-	-	-	-	-	-			
Tiamulin			-	1	7	3	-	-	-	-	-	-	-	-	-			
Vancomycin		-	-	-	-	-	-	10	1	-	-	-	-	-				
Quinupristin-dalfopristin		-	-	-	2	8	1	-	-	-	-	-	-	-				

The black areas are the test ranges not included in the test panels for the respective antimicrobial agents. Isolates that had no growth in any of the concentrations were given the lowest MIC value. Isolates with growth in all tested concentrations were given the next serially higher MIC value above the highest tested concentration (white number on black background). The MIC values of amoxicillin-clavulanic acid (2:1) and trimethoprim-sulfamethoxazole (1:19) are expressed as the MIC values of amoxicillin and trimethoprim, respectively. Different gray shading indicates the categories: susceptible—light gray; intermediate—middle gray; resistant—dark gray. For the clinical breakpoints used, please see Materials and Methods, Section 4.2.

**Table 4:** Minimal inhibitory concentrations of the 41 canine *S. pseudintermedius* isolates.

Antimicrobial Agent (s)	Number of Isolates for Which the MIC Value (mg/L) Is																	
	0.008	0.015	0.03	0.06	0.12	0.25	0.5	1	2	4	8	16	32	64	128	256	512	1024
Penicillin G	8	4	-	-	2	3	4	2	5	4	7	4	3	3				
Ampicillin			2	2	2	4	10	13	3	-	2	-	1	2	-			
Amoxicillin-clavulanic acid (2:1)			1	1	13	21	-	-	2	-	1	1	1	-	-			
Oxacillin		-	-	1	16	19	-	-	-	-	1	4						
Cephalothin				9	27	-	1	1	-	1	-	-	-	2	-	-		
Cefotaxime		-	-	-	1	14	21	-	-	-	1	1	-	3				
Cefoperazone				-	-	4	32	-	-	2	1	-	-	2				
Ceftiofur			-	-	2	34	-	-	-	1	1	-	-	1	2			
Florfenicol					-	-	-	-	34	7	-	-	-	-	-	-	-	
Erythromycin		-	-	-	4	29	-	-	-	-	-	-	-	8				
Tylosin				-	-	-	31	2	-	-	-	-	1	-	1	6		
Tilmicosin				-	-	-	3	31	-	-	-	1	-	-	-	6		
Clindamycin			-	7	27	1	-	-	1	-	-	1	1	-	3			
Streptomycin						-	-	-	2	20	11	-	-	-	2	2	-	4
Gentamicin					22	15	-	-	-	-	2	1	1	-	-	-	-	
Neomycin					20	13	-	-	-	-	3	5	-	-	-			
Ciprofloxacin	-	1	2	3	21	4	3	-	-	-	1	2	4					
Enrofloxacin	-	1	3	6	20	2	2	-	-	-	3	2	2					
Marbofloxacin	-	-	-	3	3	22	6	-	-	-	-	3	4					
Nalidixic acid				-	-	-	-	-	-	-	3	11	18	2	5	2		
Trimethoprim-sulfamethoxazole (1:19)		-	1	9	13	2	13	-	-	-	2	1	-	-				
Tetracycline					18	13	-	-	-	-	-	-	5	5	-	-	-	
Doxycycline				27	4	-	-	-	-	4	6	-	-	-	-	-		
Linezolid			-	-	-	-	5	35	1	-	-	-	-	-	-			
Tiamulin			-	1	22	17	1	-	-	-	-	-	-	-	-			
Vancomycin		-	-	-	-	-	6	34	1	-	-	-	-	-				
Quinupristin-dalfopristin		-	-	-	5	31	5	-	-	-	-	-	-	-				

The black areas are the test ranges not included in the test panels for the respective antimicrobial agents. Isolates that had no growth in any of the concentrations were given the lowest MIC value. Isolates with growth in all tested concentrations were given the next serially higher MIC value above the highest tested concentration (white number on black background). The MIC values of amoxicillin-clavulanic acid (2:1) and trimethoprim-sulfamethoxazole (1:19) are expressed as the MIC values of amoxicillin and trimethoprim, respectively. Different gray shading indicates the categories: susceptible—light gray; intermediate—middle gray; resistant—dark gray. For the clinical breakpoints used, please see Materials and Methods, Section 4.2.

## Data Availability

All data are presented in the text and tables.

## References

[1] Haag A.F., Fitzgerald J.R., Penadés J.R. (2019). *Staphylococcus aureus* in Animals. Microbiol. Spectr..

[2] Lynch S.A., Helbig K.J. (2021). The Complex Diseases of *Staphylococcus pseudintermedius* in Canines: Where to Next?. Vet. Sci..

[3] Guardabassi L., Schwarz S., Lloyd D.H. (2004). Pet animals as reservoirs of antimicrobial-resistant bacteria. J. Antimicrob. Chemother..

[4] Loeffler A., Lloyd D.H. (2010). Companion animals: A reservoir for methicillin-resistant *Staphylococcus aureus* in the community?. Epidemiol. Infect..

[5] Sing A., Tuschak C., Hörmansdorfer S. (2008). Methicillin-resistant *Staphylococcus aureus* in a family and its pet cat. N. Engl. J. Med..

[6] Walther B., Hermes J., Cuny C., Wieler L.H., Vincze S., Abou Elnaga Y., Stamm I., Kopp P.A., Kohn B., Witte W. (2012). Sharing more than friendship—nasal colonization with coagulase-positive staphylococci (CPS) and co-habitation aspects of dogs and their owners. PLoS ONE.

[7] Blondeau L.D., Deutscher M., Rubin J.E., Deneer H., Kanthan R., Sanche S., Blondeau J.M. (2021). Urinary tract infection in a human male patient with *Staphylococcus pseudintermedius* transmission from the family dog. J. Chemother..

[8] Hackmann C., Gastmeier P., Schwarz S., Lübke-Becker A., Bischoff P., Leistner R. (2021). Pet husbandry as a risk factor for colonization or infection with MDR organisms: A systematic meta-analysis. J. Antimicrob. Chemother..

[9] Schwarz S., Feßler A.T., Loncaric I., Wu C., Kadlec K., Wang Y., Shen J. (2018). Antimicrobial Resistance among Staphylococci of Animal Origin. Microbiol Spectr..

[10] Cain C.L. (2013). Antimicrobial resistance in staphylococci in small animals. Vet. Clin. N. Am. Small Anim. Pract..

[11] Malik S., Peng H., Barton M.D. (2005). Antibiotic resistance in staphylococci associated with cats and dogs. J. Appl. Microbiol..

[12] Kadlec K., Schwarz S. (2012). Antimicrobial resistance of *Staphylococcus pseudintermedius*. Vet. Dermatol..

[13] Weese J.S., van Duijkeren E. (2010). Methicillin-resistant *Staphylococcus aureus* and *Staphylococcus pseudintermedius* in veterinary medicine. Vet. Microbiol..

[14] Walther B., Tedin K., Lübke-Becker A. (2017). Multidrug-resistant opportunistic pathogens challenging veterinary infection control. Vet Microbiol..

[15] Ruiz-Ripa L., Simón C., Ceballos S., Ortega C., Zarazaga M., Torres C., Gómez-Sanz E. (2021). *S. pseudintermedius* and *S. aureus* lineages with transmission ability circulate as causative agents of infections in pets for years. BMC Vet. Res..

[16] Davis J.A., Jackson C.R., Fedorka-Cray P.J., Barrett J.B., Brousse J.H., Gustafson J., Kucher M. (2014). Carriage of methicillin-resistant staphylococci by healthy companion animals in the US. Lett. Appl. Microbiol..

[17] Perreten V., Kadlec K., Schwarz S., Grönlund Andersson U., Finn M., Greko C., Moodley A., Kania S.A., Frank L.A., Bemis D.A. (2010). Clonal spread of methicillin-resistant *Staphylococcus pseudintermedius* in Europe and North America: An international multicentre study. J. Antimicrob. Chemother..

[18] Kadlec K., Schwarz S., Perreten V., Grönlund Andersson U., Finn M., Greko C., Moodley A., Kania S.A., Frank L.A., Bemis D.A. (2010). Molecular analysis of methicillin-resistant *Staphylococcus pseudintermedius* of feline origin from different European countries and North America. J. Antimicrob. Chemother..

[19] Ruzauskas M., Couto N., Pavilonis A., Klimiene I., Siugzdiniene R., Virgailis M., Vaskeviciute L., Anskiene L., Pomba C. (2016). Characterization of *Staphylococcus pseudintermedius* isolated from diseased dogs in Lithuania. Pol. J. Vet. Sci..

[20] Loncaric I., Tichy A., Handler S., Szostak M.P., Tickert M., Diab-Elschahawi M., Spergser J., Künzel F. (2019). Prevalence of Methicillin-Resistant *Staphylococcus* sp. (MRS) in Different Companion Animals and Determination of Risk Factors for Colonization with MRS. Antibiotics.

[21] Saputra S., Jordan D., Worthing K.A., Norris J.M., Wong H.S., Abraham R., Trott D.J., Abraham S. (2017). Antimicrobial resistance in coagulase-positive staphylococci isolated from companion animals in Australia: A one year study. PLoS ONE.

[22] Couto N., Monchique C., Belas A., Marques C., Gama L.T., Pomba C. (2016). Trends and molecular mechanisms of antimicrobial resistance in clinical staphylococci isolated from companion animals over a 16 year period. J. Antimicrob. Chemother..

[23] CLSI (2020). Performance Standards for Antimicrobial Disk and Dilution Susceptibility Tests for Bacteria Isolated From Animals.

[24] Couto N., Belas A., Rodrigues C., Schwarz S., Pomba C. (2016). Acquisition of the *fexA* and *cfr* genes in *Staphylococcus pseudintermedius* during florfenicol treatment of canine pyoderma. J. Glob. Antimicrob. Resist..

[25] Kizerwetter-Świda M., Chrobak-Chmiel D., Kwiecień E., Rzewuska M., Binek M. (2021). Molecular characterization of high-level mupirocin resistance in methicillin-resistant staphylococci isolated from companion animals. Vet. Microbiol..

[26] Borio S., Colombo S., La Rosa G., De Lucia M., Damborg P., Guardabassi L. (2015). Effectiveness of a combined (4% chlorhexidine digluconate shampoo and solution) protocol in MRS and non-MRS canine superficial pyoderma: A randomized, blinded, antibiotic-controlled study. Vet. Dermatol..

[27] Loeffler A., Cobb M.A., Bond R. (2011). Comparison of a chlorhexidine and a benzoyl peroxide shampoo as sole treatment in canine superficial pyoderma. Vet. Rec..

[28] Jeffers J.G. (2013). Topical therapy for drug-resistant pyoderma in small animals. Vet. Clin. N. Am. Small Anim. Pract..

[29] Ortega Morente E., Fernández-Fuentes M.A., Grande Burgos M.J., Abriouel H., Pérez Pulido R., Gálvez A. (2013). Biocide tolerance in bacteria. Int. J. Food Microbiol..

[30] Schug A.R., Bartel A., Scholtzek A.D., Meurer M., Brombach J., Hensel V., Fanning S., Schwarz S., Feßler A.T. (2020). Biocide susceptibility testing of bacteria: Development of a broth microdilution method. Vet. Microbiol..

[31] Langsrud S., Sidhu M., Heir E., Holck A. (2003). Bacterial disinfectant resistance—A challenge for the food industry. Int. Biodeterior. Biodegrad..

[32] Wassenaar T.M., Ussery D., Nielsen L.N., Ingmer H. (2015). Review and phylogenetic analysis of *qac* genes that reduce susceptibility to quaternary ammonium compounds in *Staphylococcus* species. Eur. J. Microbiol. Immunol..

[33] CLSI (2021). Performance Standards for Antimicrobial Susceptibility Testing.

[34] CLSI (2019). Understanding Susceptibility Test Data as a Component of Antimicrobial Stewardship in Veterinary Settings Performance.

[35] Schwarz S., Silley P., Simjee S., Woodford N., van Duijkeren E., Johnson A.P., Gaastra W. (2010). Assessing the antimicrobial susceptibility of bacteria obtained from animals. Vet. Microbiol..

[36] CLSI (2021). Generation, Presentation, and Application of Antimicrobial Susceptibility Data for Bacteria of Animal Origin, a Report.

[37] Gómez-Sanz E., Kadlec K., Feßler A.T., Zarazaga M., Torres C., Schwarz S. (2013). A novel *fexA* variant from a canine *Staphylococcus pseudintermedius* isolate that does not confer florfenicol resistance. Antimicrob. Agents Chemother..

[38] Feßler A., Scott C., Kadlec K., Ehricht R., Monecke S., Schwarz S. (2010). Characterization of methicillin-resistant *Staphylococcus aureus* ST398 from cases of bovine mastitis. J. Antimicrob. Chemother..

[39] Schwarz S., Alesík E., Werckenthin C., Grobbel M., Lübke-Becker A., Wieler L.H., Wallmann J. (2007). Antimicrobial susceptibility of coagulase-positive and coagulase-variable staphylococci from various indications of swine, dogs and cats as determined in the BfT-GermVet monitoring program 2004–2006. Berl. Münch. Tierärztl. Wochenschr..

[40] Walther B., Wieler L.H., Vincze S., Antão E.M., Brandenburg A., Stamm I., Kopp P.A., Kohn B., Semmler T., Lübke-Becker A. (2012). MRSA variant in companion animals. Emerg. Infect. Dis..

[41] Boerlin P., Burnens A.P., Frey J., Kuhnert P., Nicolet J. (2001). Molecular epidemiology and genetic linkage of macrolide and aminoglycoside resistance in *Staphylococcus intermedius* of canine origin. Vet. Microbiol..

[42] EMEA (1997). Commitee for Veterinary Medicinal Products Benzalkonium Chloride Summary Report. The European Agency for the Evaluation of Medicinal Products. EMEA/MRL/306/97. https://www.ema.europa.eu/en/documents/mrl-report/benzalkonium-chloride-summary-report-committee-veterinary-medicinal-products_en.pdf.

[43] Scholtzek A.D., Hanke D., Walther B., Eichhorn I., Stöckle S.D., Klein K.S., Gehlen H., Lübke-Becker A., Schwarz S., Feßler A.T. (2019). Molecular Characterization of Equine *Staphylococcus aureus* Isolates Exhibiting Reduced Oxacillin Susceptibility. Toxins.

[44] Monecke M., Feßler A.T., Burgold-Voigt S., Krüger H., Mühldorfer K., Wibbelt G., Liebler-Tenorio E.M., Reinicke M., Braun S.D., Hanke D. (2021). *Staphylococcus aureus* isolates from Eurasian Beavers (*Castor fiber*) carry a novel phage-borne bicomponent leukocidin related to the Panton-Valentine leukocidin. Sci. Rep..

[45] Roberts M.C., Feßler A.T., Monecke S., Ehricht R., No D., Schwarz S. (2018). Molecular Analysis of Two Different MRSA Clones ST188 and ST3268 From Primates (*Macaca* spp.) in a United States Primate Center. Front Microbiol..

[46] Walther B., Klein K.S., Barton A.K., Semmler T., Huber C., Merle R., Tedin K., Mitrach F., Lübke-Becker A., Gehlen H. (2018). Equine Methicillin-Resistant Sequence Type 398 *Staphylococcus aureus* (MRSA) Harbor Mobile Genetic Elements Promoting Host Adaptation. Front. Microbiol..

[47] Murugaiyan J., Walther B., Stamm I., Abou-Elnaga Y., Brueggemann-Schwarze S., Vincze S., Wieler L.H., Lübke-Becker A., Semmler T., Roesler U. (2014). Species differentiation within the *Staphylococcus intermedius* group using a refined MALDI-TOF MS database. Clin. Microbiol. Infect..

[48] Schnellmann C., Gerber V., Rossano A., Jaquier V., Panchaud Y., Doherr M.G., Thomann A., Straub R., Perreten V. (2006). Presence of new *mecA* and *mph*(C) variants conferring antibiotic resistance in *Staphylococcus* spp. isolated from the skin of horses before and after clinic admission. J. Clin. Microbiol..

[49] Strommenger B., Kettlitz C., Werner G., Witte W. (2003). Multiplex PCR assay for simultaneous detection of nine clinically relevant antibiotic resistance genes in *Staphylococcus aureus*. J. Clin. Microbiol..

[50] Shore A.C., Deasy E.C., Slickers P., Brennan G., O’Connell B., Monecke S., Ehricht R., Coleman D.C. (2011). Detection of staphylococcal cassette chromosome *mec* type XI carrying highly divergent *mecA*, *mecI*, *mecR1*, *blaZ*, and *ccr* genes in human clinical isolates of clonal complex 130 methicillin-resistant *Staphylococcus aureus*. Antimicrob. Agents Chemother..

[51] Pang Y., Bosch T., Roberts M.C. (1994). Single polymerase chain reaction for the detection of tetracycline-resistant determinants Tet K and Tet L. Mol. Cell. Probes.

[52] Roberts M.C., Pang Y., Riley D.E., Hillier S.L., Berger R.C., Krieger J.N. (1993). Detection of Tet M and Tet O tetracycline resistance genes by polymerase chain reaction. Mol. Cell. Probes.

[53] Lüthje P., Schwarz S. (2007). Molecular basis of resistance to macrolides and lincosamides among staphylococci and streptococci from various animal sources collected in the resistance monitoring program BfT-GermVet. Int. J. Antimicrob. Agents.

[54] Li J., Li B., Wendlandt S., Schwarz S., Wang Y., Wu C., Ma Z., Shen J. (2014). Identification of a novel *vga*(E) gene variant that confers resistance to pleuromutilins, lincosamides and streptogramin A antibiotics in staphylococci of porcine origin. J. Antimicrob. Chemother..

[55] Hauschild T., Vuković D., Dakić I., Jezek P., Djukić S., Dimitrijević V., Stepanović S., Schwarz S. (2007). Aminoglycoside resistance in members of the *Staphylococcus sciuri* group. Microb. Drug Resist..

[56] López M., Kadlec K., Schwarz S., Torres C. (2012). First detection of the staphylococcal trimethoprim resistance gene *dfrK* and the *dfrK*-carrying transposon Tn*559* in enterococci. Microb. Drug Resist..

[57] Cattoir V., Huynh T.M., Bourdon N., Auzou M., Leclercq R. (2009). Trimethoprim resistance genes in vancomycin-resistant *Enterococcus faecium* clinical isolates from France. Int. J. Antimicrob. Agents.

[58] Krumperman P.H. (1983). Multiple antibiotic resistance indexing of *Escherichia coli* to identify high-risk sources of fecal contamination of foods. Appl. Environ. Microbiol..

